# Multi-Analysis Characterization of *Makgeolli* Made from the Novel Glutinous Rice Cultivar ‘*Gureumchal*’: Free Amino Acids, GC–MS Volatiles, and Electronic Tongue-Derived Flavor Profile

**DOI:** 10.3390/foods15030586

**Published:** 2026-02-05

**Authors:** Su-Hyeon Heo, Su-Hyun Lee, Jong-Hyeon Lee, Jungmin Kang, Yeonghun Kim, Hyun Mo Jung, Myung Hee Lee, Jeong-Seok Cho, Sae-Byuk Lee

**Affiliations:** 1 School of Food Science and Biotechnology, Kyungpook National University, Daegu 41566, Republic of Korea; sewt1024@korea.kr (S.-H.H.); witc54@naver.com (S.-H.L.); youni414@naver.com (J.-H.L.); jungmin3q2@naver.com (J.K.); younghun0320@naver.com (Y.K.); 2 Gyeongsangbuk-do Provincial Agricultural Research & Extension Services, Daegu 41404, Republic of Korea; 3 Division of High-Tech Agricultural Industry, Kyongbuk Science University, Chilgok 39913, Republic of Korea; hmjung@kbsu.ac.kr (H.M.J.); mhlee@kbsc.ac.kr (M.H.L.); 4 Smart Food Manufacturing Research Group, Korea Food Research Institute, Wanju-gun 55365, Republic of Korea; 5 Institute of Fermentation Biotechnology, Kyungpook National University, Daegu 41566, Republic of Korea

**Keywords:** glutinous rice, *Makgeolli*, free amino acids, volatile aromatic compounds, E-tongue

## Abstract

This study evaluated the suitability of a new glutinous rice cultivar of *Gureumchal* as a raw material for *Makgeolli*, a traditional Korean rice wine, by comparing *Makgeolli* produced from *Gureumchal* with those made from a non-glutinous rice and another glutinous rice cultivar. *Makgeolli* was prepared using single and blended rice combinations, and their physicochemical characteristics, amino acids, volatile aromatic compounds, and E-tongue were analyzed. The *Gureumchal* produced generally higher levels of total amino acids and ester compounds, particularly fruity esters, when compared with the other rice formulations. A volatile aromatic compound analysis indicated that non-glutinous rice favored the formation of acetate esters typically associated with the acetyl-CoA pathway, whereas *Gureumchal* produced higher levels of fruity acyl-CoA-derived esters, such as ethyl hexanoate and ethyl octanoate. An E-tongue analysis further demonstrated that rice type strongly shaped the *Makgeolli*’s taste profile: glutinous rice samples, including *Gureumchal*, exhibited higher sweetness but low umami, whereas non-glutinous rice produced higher acidity and umami. Blended samples confirmed that manipulating the proportion of glutinous and non-glutinous rice allows the systematic adjustment of taste balance. Overall, *Gureumchal* formed a distinct flavor profile characterized by fruity esters and pronounced sweetness, indicating its potential to diversify *Makgeolli* quality and support targeted flavor design.

## 1. Introduction

*Makgeolli* is a traditional Korean alcoholic beverage produced by fermenting starchy raw materials, including non-glutinous rice, wheat, or sweet potatoes, with a diverse range of microorganisms such as yeasts, molds, and lactic acid bacteria (LAB) is characterized by the metabolic interactions among these microbes during fermentation, which collectively determine its distinctive characteristics [1,2]. In the saccharification stage, amylases hydrolyze starch into fermentable sugars, which are subsequently converted into ethanol by yeast [3,4]. Through this process, diverse bioactive compounds and *Makgeolli*’s characteristic aroma are generated [5].

Rice (*Oryza sativa* L.) is the most commonly used raw material in *Makgeolli* production, and the chemical composition of the rice used strongly influences microbial fermentation kinetics and flavor formation [6,7]. The primary constituents of *Makgeolli* include carbohydrates, proteins, lipids, organic acids, minerals, free amino acids (FAAs), and volatile aroma compounds [8,9,10]. The starch composition of the rice determines saccharification efficiency and fermentation behavior. Non-glutinous rice, the type typically used for *Makgeolli*, primarily consists of amylose and amylopectin, whereas glutinous rice is almost entirely composed of amylopectin [11,12]. These differences influence gelatinization, viscosity, and saccharification efficiency [13]. Moreover, glutinous rice contains higher levels of protein, polyphenols, and other functional components than non-glutinous rice, which is beneficial for the growth of certain microorganisms, such as lactic acid bacteria [14]. Kee et al. [15] reported that *Makgeolli* containing glutinous rice exhibited enhanced sweetness and flavor intensity when compared with samples made solely from non-glutinous rice. During fermentation, rice proteins are hydrolyzed into FAAs, which serve as nitrogen sources for yeasts. Additionally, some amino acids are metabolized through the Ehrlich pathway to form higher alcohols, such as isoamyl alcohol and phenylethyl alcohol [16]. These higher alcohols can further react via the acyl-CoA pathway through alcohol acetyltransferase to generate esters, such as isoamyl acetate and phenylethyl acetate, which contribute fruity and floral notes to the *Makgeolli* [17,18]. In addition, FAAs also directly influence taste attributes, including sweetness, umami, and bitterness, contributing to overall flavor perception [19].

Recent efforts to improve the quality of *Makgeolli* have focused on (i) selecting yeast and LAB strains for co-inoculation, (ii) optimizing milling and steaming conditions, (iii) controlling fermentation temperature and duration, and (iv) enhancing distribution and storage stability [11,20,21,22,23,24,25]. In addition, state-of-the-art analytical approaches increasingly rely on integrated instrumental strategies that combine electronic sensory tools (E-nose/E-tongue) with chromatographic profiling (GC–MS and GC–IMS) and multivariate statistical analysis to link processing variables with chemical and sensory attributes and to enhance discrimination of product quality [26,27,28].

However, most studies have focused on *Makgeolli* produced from non-glutinous rice, and systematic evaluations of glutinous rice varieties with distinct starch structures remain limited. In particular, *Gureumchal* (Gyeongbuk No. 13), a glutinous rice cultivar recently developed in Gyeongsangbuk-do, is known for its relatively low carbohydrate content, unique starch composition, and excellent aroma when steamed. Based on these qualities, *Gureumchal*’ may impart a distinct FAA and volatile profile to *Makgeolli*, potentially enhancing fruity and floral attributes. Yet, its fermentation dynamics and volatile aromatic compositions during *Makgeolli* production have not been sufficiently explored.

This study investigated the fermentation characteristics, FAA composition, and volatile aroma profile of *Makgeolli* produced using the ‘*Gureumchal*’ glutinous rice variety. By integrating data from GC–MS, E-nose, and E-tongue analyses, we sought to link chemical composition with taste-related attributes in a comprehensive manner. By comparing this ‘*Gureumchal*’ *Makgeolli* with *Makgeolli* made from non-glutinous rice, this work demonstrates the potential of ‘*Gureumchal*’ as a high-value raw material for traditional liquor production and contributes to the diversification and modernization of the *Makgeolli* industry.

## 2. Materials and Methods

### 2.1. Materials, Samples, and Strains

‘*Baekjinju*’ rice harvested in 2024 was used as the non-glutinous rice cultivar (Gyeonggi-mi, Hyundai Nongsan, Anseong, Republic of Korea). Two glutinous rice cultivars, *Gureumchal*’ and ‘*Baegokchal*’, were provided by Gyeongsangbuk-do Agricultural Research & Extension Services (Daegu, Republic of Korea). An *Aspergillus luchuensis*-inoculated rice koji produced by Suwon Fermentation (Suwon, Republic of Korea) was used as the starter. Brewing water was a commercial bottled water (Tamsasoo; Sansu, Namyangju, Republic of Korea). The yeast was the commercial *Saccharomyces cerevisiae* Fermivin (DSM Food Specialties, Heerlen, The Netherlands).

### 2.2. Makgeolli Fermentation

A seed mash was prepared by mixing 86 g of rice koji with 172 mL of water, inoculating the mixture with *S. cerevisiae* Fermivin dry yeast at 0.02% (*w*/*v*), and then incubating it at 25 °C for 48 h. For the main mash (first-stage fermentation), 1314 g of steamed rice, 400 g of rice koji (sp 100), and 3428 g of water were combined with the seed mash. For blended formulations, cultivar ratios were prepared on a polished rice weight basis (*w*/*w*, prior to steaming), and the rice was then steamed under identical conditions. Sample names were defined by cultivar composition (*w*/*w*): C100 (100% *Baekjinju*, non-glutinous), G100 (100% *Gureumchal*, glutinous), B100 (100% *Baegokchal*, glutinous), C60G40 (60% *Baekjinju* + 40% *Gureumchal*, *w*/*w*), and C80G20 (80% *Baekjinju* + 20% *Gureumchal*, *w*/*w*). Fermentation was conducted for 5 days in a fermentation room at 20 °C. Once fermentation was completed, the *Makgeolli* was filtered through a 40-mesh (0.4 mm holes) cloth and centrifuged at 3578× *g* and 4 °C for 10 min to remove the solid residues and for further analysis of various characteristics and components. Fermentation for each formulation was performed as a single independent batch. All analytical measurements were performed in technical triplicate using the same batch.

### 2.3. Analysis of Quality Characteristics 

The fermentation properties of *Makgeolli* were analyzed using a supernatant obtained by centrifugation at 3578× *g* for 15 min. Soluble solids were then measured using a refractometer (RA 250, ATAGO, Tokyo, Japan), pH was measured using a pH meter (a-AB33PH, Ohaus Co., Parsippany, NJ, USA), and total acidity was determined by titrating the filtrates with 0.1 N NaOH (expressed as g/L of citric acid) [29]. To determine the alcohol content, 100 mL of the supernatant was transferred to a distillation flask. The remaining residue in the measuring flask was rinsed twice with 15 mL of distilled water, with the resulting rinse water added to the distillation flask. Subsequently, the mixture was distilled to obtain 70 mL of distillate. The alcohol content of the mixed distillate was measured using a hydrometer based on the specific gravity (expressed as % [*v*/*v*]), and the temperature was corrected to 15 °C using the Gay–Lussac alcoholometric table [29]. The concentrations of free sugars and organic acids were determined by HPLC (Model Prominence, Shimadzu, Kyoto, Japan) with a Sugar-Pak I column (diameter 6.5 × 300 mm; Waters, Milford, MA, USA) and a PL Hi-Plex H column (diameter 7.7 × 300 mm; Agilent Technologies, Santa Clara, CA, USA), respectively. For free sugars, the chromatography conditions included a flow rate of 0.5 mL/min and a temperature of 90 °C, with 50 mg/L Ca-ethylenediaminetetraacetic acid (Ca-EDTA) buffer as the mobile phase [30]. For organic acids, conditions included a flow rate of 0.6 mL/min and temperature of 65 °C, with 0.005 mol sulfuric acid as the mobile phase. For both, detection was performed using a refractive index detector (RID-10A, Shimadzu) [31].

### 2.4. Amino Acid Characterization

Samples were hydrolyzed at 110 °C for 24 h under reduced nitrogen pressure by adding 6 N HCl. The amino acid content in the hydrolysate was then analyzed using an amino acid autoanalyzer (L-8900 Model, Hitachi, Tokyo, Japan). The results were compared with the retention times of the amino acid standards, and content levels were determined based on peak area.

### 2.5. Volatile Aromatic Compound Analysis

Volatile aromatic compounds in the *Makgeolli* were analyzed using an Agilent 8890 gas chromatograph coupled with a 5977C mass spectrometer (Agilent Technologies Inc., Santa Clara, CA, USA). Volatile aromatic compounds were extracted through headspace-solid phase microextraction (HS-SPME) with a 50/30 μm DVB/CAR/PDMS fiber (Supelco, Bellefonte, PA, USA) in headspace mode following the method described by Lee et al. [32]. For sample preparation, 5 mL of the supernatant was transferred to a 20 mL headspace vial (PTFE/silicon septum; magnetic cap) containing 1.25 g of NaCl to increase ionic strength and enhance the release of volatiles aromatic compounds into the headspace. The vial was sealed and equilibrated at a 35 °C for 20 min with agitation (150 rpm). Then, the SPME fiber was exposed to the headspace at 35 °C for 40 min to adsorb volatile aromatic compounds. The thermally desorbed in the GC inlet at 250 °C for 2 min in split mode. Chromatographic separation was carried out on a DB-WAX column (60 m × 250 μm × 0.25 mm, Agilent Technologies). The GC oven temperature program began at 40 °C, which was held for 2 min, increased first to 220 °C at a rate of 2 °C/min and then to 240 °C at 20 °C/min, with a final hold at 240 °C for 5 min. The carrier gas consisted of pure helium (99.999%) at a flow rate of 1 mL/min. Volatile aromatic compounds were tentatively identified by matching EI mass spectra against the NIST 23 Mass Spectral Library (John Wiley and Sons, Inc., Hoboken, NJ, USA). Identification was further supported by linear retention indices (LRIs), which were calculated using a homologous series of n-alkanes (C7–C30) analyzed under the same GC conditions. Compounds were accepted only when both criteria were satisfied: a spectral similarity score ≥ 70% and RI difference lower than 30 were chosen for further analysis. Volatile aromatic compounds were semi-quantitatively estimated using an area-based approach relative to authentic standards (MSIGCL412, MetaSci, Toronto, ON, Canada; purity ≥ 99.9%) analyzed under the same conditions and were reported as estimated concentrations (mg/L).

### 2.6. E-Tongue

Taste profiles of the *Makgeolli* samples were evaluated using an E-tongue system (ASTREE, Alpha MOS, France). Each sample was analyzed with seven sensors (AHS for sourness-related; CTS for saltiness-related; NMS for umami-related; ANS for sweetness-related; SCS for bitterness-related, with two reference electrodes, a PKS and CPS). These sensors do not directly quantify individual chemical compounds but rather provide overall taste perceptions expressed as sensor response intensities ranging from 0 to 12, allowing the relative interpretation of taste profiles. For sample preparation, *Makgeolli* was centrifuged at 3000 rpm for 5 min, and the supernatant was used for analysis. Each measurement was performed for 120 s under identical acquisition conditions. The obtained response data were converted into relative sensor scores corresponding to each sensor’s sensitivity range, enabling the visualization of characteristic taste patterns. Statistical evaluation and visualization of the E-tongue data were performed using the AlphaSoft software package (Version 7.3.0; Alpha MOS, Toulouse, France). The relative sensor scores were visualized as radar charts to compare instrumental taste patterns among formulations.

### 2.7. Statistical Analysis

All analytical measurements for experiments were performed at least triplicate (*n* = 3), and results were expressed as the mean ± standard deviation (SD). Statistical analyses were performed using SAS software (Version 9.4; SAS Institute Inc., Cary, NC, USA). Principal component analysis (PCA) and figure preparation were performed using GraphPad Prism (version 10.4.1 for Windows, GraphPad Software, San Diego, CA, USA).

## 3. Results and Discussion

### 3.1. Physicochemical Characteristics of Makgeolli Made Using Different Rice Cultivars and Cultivar Blending Ratios

The physicochemical properties of the tested *Makgeolli* are presented in Table 1. The soluble solid content was slightly lower in the *Makgeolli* made with *Gureumchal* (G100) than in those produced using non-glutinous rice or *Baegokchal* (B100), and the reducing sugar content ranged from 0.22% to 0.32%, indicating that fermentation proceeded smoothly. The alcohol content was slightly higher in the B100 and C80G20 (8.80%) *Makgeolli* samples, whereas G100 exhibited the lowest level, 8.40%. The pH levels ranged from 3.23 to 3.52, and the total acidity was lowest, at 0.49%, in *Makgeolli* made with *Gureumchal* (G100) and gradually increased as the proportion of non-glutinous rice in the blend increased. Two organic acids, citric acid and lactic acid were detected, with no significant differences in concentration among *Makgeolli* samples. In the free sugar analysis, glucose content was significantly lower in the G100 (40.44 mg/mL) *Makgeolli* than in those made from other rice varieties, and it increased when non-glutinous rice was incorporated. Fructose content was significantly higher in *Makgeolli* made with *Baegokchal* than in *Makgeolli* produced from non-glutinous rice or *Gureumchal*, whereas the C60G40 blend produced fructose levels similar to those of non-glutinous rice *Makgeolli*. Overall, except for total acidity and the free sugar composition, differences among rice varieties did not substantially affect the physicochemical properties of *Makgeolli*. The variations observed in total acidity and sugar content are likely attributable to differences in the nutritional composition of the rice varieties, which influence fermentation behavior.

The lactic acid in *Makgeolli* is primarily produced by the metabolism of lactic acid bacteria (LAB) and is strongly influenced by environmental factors, such as fermentation temperature, oxygen availability (aerobic or anaerobic conditions), and the composition of the initial microbial community [33]. In this study, all samples were fermented under identical conditions, and no significant differences in lactic acid content were observed, indicating that alcoholic fermentation rather than lactic acid fermentation predominated in all samples. However, significant differences were observed in the free sugar and alcohol contents, soluble solids, etc., among the samples, which are likely attributable to differences in starch structure between the glutinous and non-glutinous rice types as well as varietal differences among the rice cultivars [34]. Glutinous rice, which is predominantly composed of amylopectin, undergoes rapid gelatinization, allowing saccharifying enzymes, such as α-amylase and glucoamylase, to readily access starch granules, thereby promoting efficient saccharification. In contrast, non-glutinous rice has been reported to have a high amylose content, which inhibits starch swelling and leads to the formation of a dense gel structure upon cooling, thereby limiting enzyme penetration and reducing saccharification efficiency [35].

Notably, the fermentation pattern observed in this study partially deviated from expectations based on starch structure. *Makgeolli* G100 showed the lowest residual sugar content, indicating relatively efficient saccharification and alcoholic fermentation. Nevertheless, it also exhibited the lowest alcohol content among the samples. In contrast, the C100 and B100 *Makgeolli* exhibited relatively high alcohol content, despite retaining higher levels of residual sugars at the end of fermentation. These results are partly consistent with the previous study of Wang et al. [34], which showed that glutinous rice-based *Makgeolli* tended to have a lower alcohol contents than those derived from non-glutinous rice. However, in the present experiment, the glutinous rice *Baegokchal* produced the highest alcohol content, suggesting a strong influence of cultivar-specific characteristics on fermentation performance. Overall, physicochemical differences observed among the *Makgeolli* samples appear to result from the combined effects of multiple factors, including fermentation conditions, differences in saccharification efficiency, yeast metabolic interactions, and the starch structure and other cultivar-specific characteristics of rice used. These factors appear to play decisive roles in determining *Makgeolli* quality and flavor formation.

### 3.2. Free Amino Acids of Makgeolli Made Using Different Rice Cultivars and Cultivar Blending Ratios

**Figure 1:**
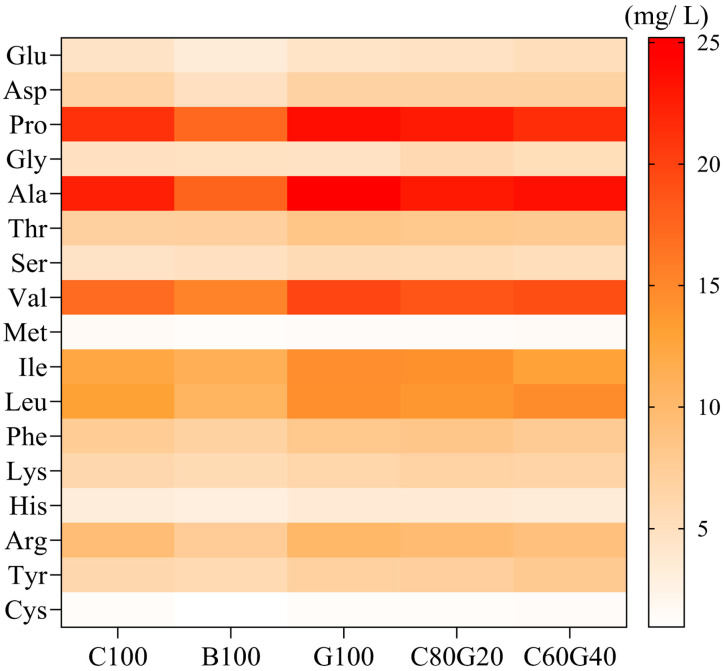
Heatmap of free amino acid contents in different *Makgeolli* made from different rice cultivars and cultivar blends.

Seventeen free amino acids were detected in the *Makgeolli* samples, and their concentrations varied markedly depending on the rice cultivar and blending ratio used (Figure 1 and Table 2). The total amino acid content was highest in *Makgeolli* produced exclusively with *Gureumchal* rice (164.88 ± 2.16 mg/L), followed by those made from non-glutinous rice (148.78 ± 0.84 mg/L) and *Baegokchal* rice (127.87 ± 1.02 mg/L). *Makgeolli* produced using blends of *Gureumchal* and non-glutinous rice exhibited amino acid levels between those produced using the constituent cultivars alone (160.95 ± 2.12 mg/L for C80G20 and 158.87 ± 2.69 mg/L for C60G40). *Makgeolli* G100 exhibited elevated levels of umami-related amino acids but also substantially higher concentrations of bitter-related amino acids. In contrast, *Makgeolli* produced with a 6:4 non-glutinous and *Gureumchal* rice blend maintained high levels of umami-related amino acids while bitter-related amino acid levels dropped relative to those seen in G100. These results suggest that blended formulations may offer an improved overall taste profile when compared to *Makgeolli* made solely from *Gureumchal*.

During fermentation, changes in free amino acid contents reflect their roles: they serve as the precursors of volatile aromatic compounds produced via the Ehrlich pathway and at the same time, are depleted through nitrogen assimilation by yeasts, enzymatic reactions, and Strecker degradation [36,37]. Additionally, as individual amino acids contribute differently to taste, changes in their concentrations can influence the sensory properties of *Makgeolli* [38], either degrading or enhancing the overall flavor complexity. Zhao et al. [39] reported that Hakka rice wine fermented from a mixture of indica non-glutinous and glutinous rice had relatively low free amino acid contents but exhibited an improved volatile aroma profile.

Gong et al. [14] reported that, when the amino acid contents of Indica and Japonica rice types were compared, the Japonica glutinous variety contained higher amounts than Indica rice. These studies suggest that fermentation using *Gureumchal*, the glutinous Japonica rice cultivar used in the present experiment, could improve the volatile aromatic compound profile in the product. We demonstrated that including 20–40% *Gureumchal* in the rice blend is a practical fermentation strategy that enhances *Makgeolli* quality and flavor formation by controlling the production and supply of free amino acids. However, the amino acid composition of the raw rice was not directly determined; therefore, this study is limited in its ability to fully elucidate the fermentation mechanisms responsible for the observed differences in amino acid contents. Future studies will be required to more clearly elucidate the relationship between amino acid compositions and contents and the flavor characteristics of the *Makgeolli.*

### 3.3. Volatile Aromatic Compounds in the Tested Makgeolli

**Figure 2:**
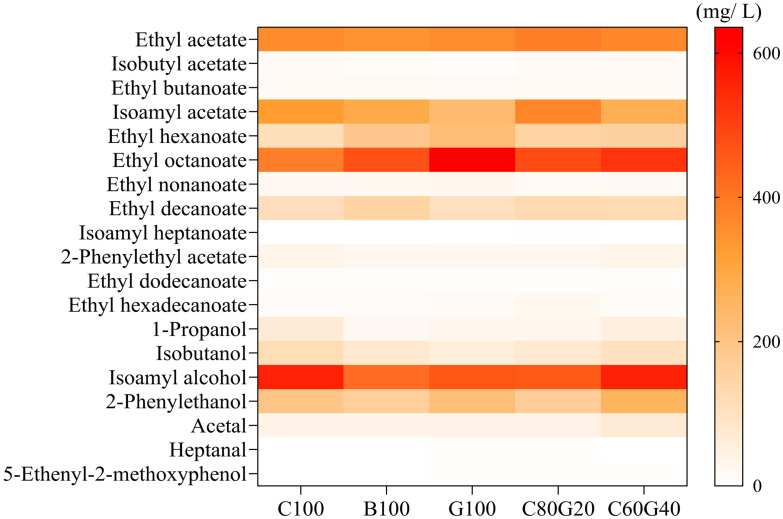
Heatmap showing the concentrations (mg/L) of volatile aromatic compounds in *Makgeolli* made using different rice cultivars and cultivar blends.

Through GC–MS analysis, a total of 19 volatile aromatic compounds were identified in the *Makgeolli* samples: 12 esters, 4 alcohols, 2 aldehydes, and 1 phenolic (Table 3). All samples shared similar volatile aromatic compound profiles. However, quantitative compositions differed markedly depending on the rice cultivars and blending ratios (Figure 2).

In this study, no statistically significant differences in ethyl acetate content were observed among the samples, while the levels of isoamyl acetate, 2-phenylethyl acetate, ethyl hexanoate, and ethyl octanoate, among others, differed significantly. Isoamyl acetate was present at relatively high levels in all samples, with *Makgeolli* C80G20 showing the highest (373.42 ± 15.30 mg/L). In contrast, *Makgeolli* G100 exhibited the lowest isoamyl acetate content (233.90 ± 13.74 mg/L). Other acetate-derived ester levels were also relatively low in the G100 samples, but the total ester content was highest, reaching 1657.61 ± 30.07 mg/L. This pattern may be driven primarily by increased levels of ethyl hexanoate and ethyl octanoate in this sample. In *Makgeolli* C100, which was fermented using only non-glutinous rice, the ethyl hexanoate content was 113.11 ± 4.44 mg/L, whereas that of G100 was markedly higher at 223.28 ± 9.54 mg/L, and ethyl octanoate exhibited a similar trend. Conversely, alcohol compound contents displayed the opposite pattern. Specifically, C100 exhibited high levels of the higher alcohols isobutanol and isoamyl alcohol, which may have contributed to acetate ester formation via acetyl-CoA–related routes, consistent with higher acetate-related ester levels [40]. On the other hand, the G100 and C60G40 samples contained high 2-phenylethanol levels of 219.69 ± 1.41 and 257.51 ± 13.17 mg/L, respectively. Although C80G20 exhibited a lower level of 2-phenylethanol (169.13 ± 5.82 mg/L), the higher proportion of *Gureumchal* in C60G40 appeared to partially enhance 2-phenylethanol production compared with C100. Nevertheless, these differences are more likely attributable to variations in yeast fermentation metabolism along the Ehrlich pathway [38].

**Figure 3:**
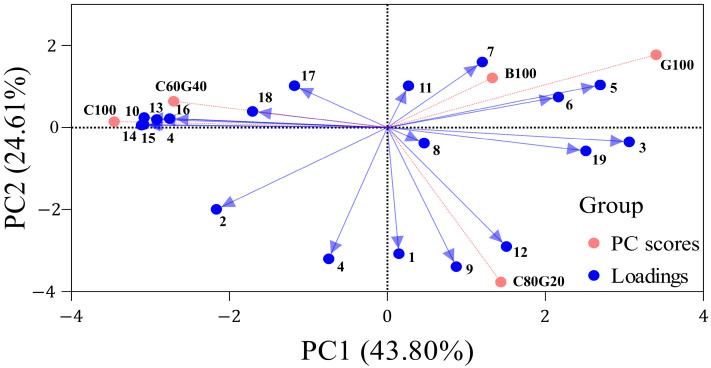
Biplot of variable loadings and principal component scores from the principal component analysis of volatile aromatic compounds. Numbers represent specific compounds: 1, ethyl acetate; 2, isobutyl acetate; 3, ethyl butanoate; 4, isoamyl acetate; 5, ethyl hexanoate; 6, ethyl octanoate; 7, ethyl nonanoate; 8, ethyl decanoate; 9, isoamyl heptanoate; 10, 2-phenylethyl acetate; 11, ethyl dodecanoate; 12, ethyl hexadecanoate; 13, 1-propanol; 14, isobutanol; 15, isoamyl alcohol; 16, 2-phenylethanol; 17, acetal; 18, heptanal; and 19, 5-ethenyl-2-methoxypheonol.

A principal component analysis (PCA) based on the volatile aromatic compound concentrations revealed clear clustering patterns that distinguished the samples from one another (Figure 3). The PCA explained 67.11% of the total variance in the first two component axes, and the single-cultivar samples (C100, B100, and G100) formed clearly separate clusters, confirming that the volatile aroma profiles of *Makgeolli* differed substantially depending on the rice cultivar used to create it. In addition, clear differences were observed among the *Makgeolli* samples produced by blending the *Gureumchal* and non-glutinous rice (C80G20 and C60G40).

Volatile aromatic compounds formed during *Makgeolli* fermentation largely originate from yeast metabolic processes and can vary depending on the degree of starch gelatinization and the availability of fermentable sugars, amino acids, fatty acids, and other compounds [33]. Chun et al. [35] found that the high amylose content of non-glutinous rice results in insufficient starch gelatinization for proper *Makgeolli* fermentation, which can also directly influence the yeast fermentation metabolic pathways. Among volatile aromatic compounds, esters are low-threshold substances that impart floral and fruity aromas. They are synthesized through two pathways: (1) the acetyl-CoA pathway, in which acetate esters are formed from higher alcohols, and (2) the acyl-CoA pathway, in which alcohols react with acyl-CoA to produce ethyl esters [40].

In this study, the observed ester pattern in G100 higher ethyl esters with relatively lower acetate esters is consistent with differences in yeast ester formation routes (e.g., acyl-CoA pathway versus acetyl-CoA pathway); however, pathway-level causality was not directly assessed in this study. A similar pattern was observed in B100, suggesting that this pattern reflects cultivar-dependent differences affecting the yeast’s fermentation metabolism. Ethyl hexanoate and ethyl octanoate are esters known to be formed through the degradation of fatty acids [41], and rice is rich in linoleic acid (C18:2) and contains substantial amounts of linolenic acid (C18:3), providing a strong potential precursor pool for ester production via the LOX–HPL pathway [42]. Thus, the differences in ester contents produced during fermentation seen in the current study can also likely be explained by cultivar differences in fatty acid release, which in turn affect ester formation. However, as the fatty acid composition of rice differs according to cultivar and production area, additional analysis of the fatty acids in the rice types used in this study would be required to fully clarify these effects [43].

The amino acid–derived volatile aromatic compounds isoamyl acetate and isobutyl acetate are produced through the Ehrlich pathway. The precursor amino acids L-leucine and L-valine are converted into α-keto acids and subsequently aldehydes through various enzymatic reactions, after which they are reduced to isoamyl alcohol and isobutanol by alcohol dehydrogenase. These fusel alcohols are then esterified via the alcohol acetyltransferase pathway by acetyl-CoA to form the corresponding acetate esters [33,38]. In this study, C100 exhibited high levels of isoamyl alcohol and isobutanol, suggesting that amino acids were sufficiently metabolized through the Ehrlich pathway during fermentation. Phenylalanine, another major precursor amino acid associated with aroma-producing compounds, is also metabolized via the same pathway, yielding 2-phenylethanol and 2-phenylethyl acetate. Most fusel alcohols were present at their highest levels in C100; however, 2-phenylethanol increased moderately when *Gureumchal* was added, further supporting the idea that rice cultivar differences affect yeast fermentation metabolism.

According to a study by Shen et al. [44], rice wines produced from non-glutinous rice tended to contain higher levels of acetate esters than those made from glutinous rice. This pattern may be partly attributed to a more efficient release of amino acids during the fermentation of non-glutinous rice. However, since the protein contents and amino acid compositions of both glutinous and non-glutinous rice vary with cultivar and production region, further investigations analyzing the amino acid profiles of raw rice from the cultivars used in this study in conjunction with measured yeast metabolic responses during fermentation will be needed to fully elucidate these relationships [45,46].

**Table 3:** Volatile aromatic compound contents (mg/L) of *Makgeolli* made using different rice cultivars and cultivar blends.

Compound	Odor Description	RI	Threshold (mg/L)	OAV (min)	OAV (max)	C100	B100	G100	C80G20	C60G40	Identification (MS/RI)
**Esters**											
Ethyl acetate	Fruity, solvent-like	820	7.50 [47]	45.88	51.52	359.1 ± 12.9 ^a^	344.1 ± 8.0 ^a^	359.8 ± 28.1 ^a^	386.4 ± 30.8 ^a^	364.0 ± 18.1 ^a^	MS/RI
Isobutyl acetate		1037	0.066 [48]	127.25	213.82	13.61 ± 0.97 ^a^	12.69 ± 0.65 ^a^	8.40 ± 0.37 ^b^	14.11 ± 0.58 ^a^	14.10 ± 1.23 ^a^	MS/RI
Ethyl butanoate	Fruity, sweet strawberry	1060	0.02 [47]	752.37	991.92	15.05 ± 0.89 ^c^	17.40 ± 0.75 ^abc^	19.84 ± 0.77 ^a^	18.70 ± 1.69 ^ab^	16.21 ± 0.63 ^bc^	MS/RI
Isoamyl acetate	Banana	1145	0.003 [48]	77966.93	124472.45	323.9 ± 10.4 ^b^	290.7 ± 7.4 ^c^	233.9 ± 13.7 ^d^	373.4 ± 15.3 ^a^	274.8 ± 12.1 ^c^	MS/RI
Ethyl hexanoate	Apple, peach	1259	0.014 [47,48]	8079.00	15948.63	113.1 ± 4.4 ^d^	185.2 ± 3.2 ^b^	223.3 ± 9.5 ^a^	152.3 ± 6.1 ^c^	157.62 ± 11.56 ^c^	MS/RI
Ethyl octanoate	Pineapple, pear, floral, banana	1464	0.005 [47,48]	77414.54	127158.01	387.0 ± 20.4 ^c^	471.3 ± 22.4 ^b^	635.8 ± 32.1 ^a^	486.1 ± 28.1 ^b^	526.4 ± 19.0 ^b^	MS/RI
Ethyl nonanoate	Fruity, coconut	1567	0.20 [47]	83.56	119.51	21.57 ± 0.08 ^a^	22.14 ± 1.18 ^c^	23.90 ± 2.25 ^a^	16.71 ± 0.93 ^b^	16.9 ± 1.3 ^b^	MS
Ethyl decanoate	Fruity, fatty	1671	0.20 [47,48]	546.08	750.43	111.4 ± 5.0 ^c^	150.1 ± 6.8 ^a^	109.2 ± 6.3 ^c^	129.5 ± 7.3 ^b^	126.9 ± 7.4 ^bc^	MS/RI
Isoamyl heptanoate		1691		0.00	0.00	ND	ND	ND	1.43 ± 0.06	ND	MS
2-Phenylethyl acetate	Floral	1847	0.25 [47,48]	98.12	142.09	35.52 ± 1.50 ^a^	25.60 ± 1.30 ^b^	24.53 ± 0.79 ^b^	25.60 ± 1.29 ^b^	33.97 ± 1.02 ^a^	MS/RI
Ethyl dodecanoate	Rose, honey	1879	1.50 [47]	2.01	3.86	3.44 ± 0.09 ^bc^	5.79 ± 0.18 ^a^	3.28 ± 0.17 ^cd^	3.02 ± 0.15 ^d^	3.76 ± 0.11 ^b^	MS/RI
Ethyl hexadecanoate	Wax	2296	1.50 [47]	7.52	15.40	11.27 ± 0.56 ^c^	11.37 ± 0.79 ^c^	15.69 ± 0.59 ^b^	23.10 ± 1.42 ^a^	13.05 ± 0.60 ^c^	MS/RI
Subtotal						1395 ± 10 ^c^	1536 ± 35 ^b^	1658 ± 30 ^a^	1630 ± 23 ^a^	1547 ± 28 ^b^	
**Alcohols**											
1-Propanol		1065				66.93 ± 2.45 ^a^	21.15 ± 0.88 ^d^	26.86 ± 1.56 ^c^	27.27 ± 0.92 ^c^	54.69 ± 0.26 ^b^	MS/RI
Isobutanol	Fusel, alcohol	1118	40 [47,48]	1.57	3.00	120.1 ± 3.8 ^a^	78.43 ± 3.87 ^c^	62.89 ± 1.63 ^d^	75.94 ± 3.18 ^c^	105.4 ± 4.9 ^b^	MS/RI
Isoamyl alcohol	Whiskey, malt	1235	30 [47,48]	14.09	18.71	560.0 ± 23.2 ^a^	422.6 ± 16.4 ^b^	464.9 ± 18.4 ^b^	457.3 ± 9.3 ^b^	561.3 ± 40.4 ^a^	MS/RI
2-Phenylethanol	Flowery, Rose, honey pollen	1946	14 [47,48]	11.94	18.39	197.1 ± 6.2 ^c^	167.2 ± 5.1 ^d^	219.7 ± 1.4 ^b^	169.1 ± 5.8 ^d^	257.5 ± 13.2 ^a^	MS/RI
Subtotal						979.0 ± 16.0 ^a^	714.8 ± 11.9 ^c^	798.7 ± 16.7 ^b^	756.1 ± 16.4 ^bc^	1013 ± 37 ^a^	
**Aldehydes**											
Acetal	Pungent, green, woody solvent	825				41.49 ± 1.14 ^b^	38.87 ± 1.80 ^b^	40.81 ± 0.88 ^b^	39.82 ± 1.28 ^b^	69.65 ± 5.02 ^a^	MS/RI
Heptanal		1134	0.003 [49]	0	1090	ND	ND	3.27 ± 0.17 ^a^	2.11 ± 0.10 ^b^	ND	MS
Subtotal						41.49 ± 1.14 ^b^	38.87 ± 1.80 ^b^	44.08 ± 0.81 ^b^	41.93 ± 1.19 ^b^	69.65 ± 5.02 ^a^	
**Other**											
5-Ethenyl-2- methoxyphenol		2230				ND	ND	2.16 ± 0.12 ^b^	2.04 ± 0.00 ^b^	2.38 ± 0.08 ^a^	MS
Total						2503 ± 16 ^b^	2290 ± 42 ^c^	2416 ± 27 ^b^	2430 ± 9 ^b^	2633 ± 61 ^a^	

All the data were expressed as the mean ± SD (*n* = 3). ND, not detected. In each row, values followed by different letters differ significantly (*p* < 0.05). Odor descriptions and threshold values are derived from previous studies [47,48,49,50,51,52]. Odor activity values (OAVs) were calculated by dividing the minimum and maximum observed concentrations for each compound by its odor threshold value.

Overall, during *Makgeolli* fermentation, non-glutinous rice enhanced the formation of amino acid–derived aroma compounds through the Ehrlich pathway, whereas glutinous rice promoted the production of fatty acid derived esters via the acyl-CoA pathway. In *Makgeolli* G100, in particular, ester production increased markedly, resulting in more pronounced fruity and floral aroma characteristics. When rice cultivars were blended, the isoamyl alcohol content in C60G40 samples was comparable to that in C100 samples, whereas *Makgeolli* C80G20 exhibited a higher isoamyl acetate content than the C100 samples. This phenomenon may be interpreted as a shift in the balance of metabolic pathway flux during fermentation, with a concomitant shift in aroma production patterns, driven by differences in the solubility of fermentable sugars available to the yeasts and by changes in substrate composition according to the mixing ratio. Therefore, modulating the rice cultivars and blending ratios used in *Makgeolli* production is a promising path for product improvement, and our study suggests *Gureumchal* has potential as a novel raw material for enhancing aroma quality in the *Makgeolli* market.

### 3.4. E-Tongue-Derived Flavor Profiles of the Tested Makgeolli

**Figure 4:**
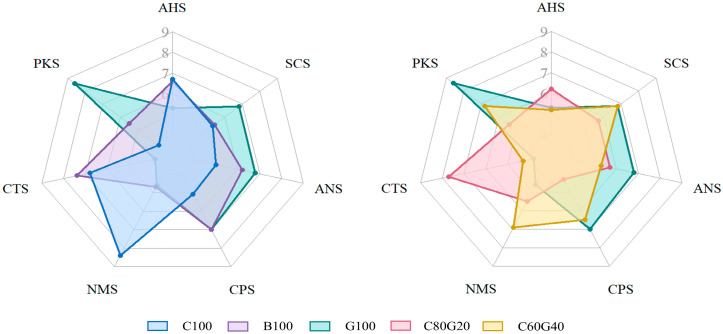
E-tongue-derived taste intensity measurements for *Makgeolli* made from different rice cultivars and cultivar blends. Radial scale indicates taste intensity (0–9). AHS for sourness-related; CTS for saltiness-related; NMS for umami-related; ANS for sweetness-related; SCS for bitterness-related, with two reference electrodes, a PKS and CPS. Sample names were defined by cultivar composition (*w*/*w*): C100 (100% *Baekjinju*, non-glutinous), G100 (100% *Gureumchal*, glutinous), B100 (100% *Baegokchal*, glutinous), C60G40 (60% *Baekjinju* + 40% *Gureumchal*, *w*/*w*), and C80G20 (80% *Baekjinju* + 20% *Gureumchal*, *w*/*w*).

E-tongue analysis showed that the instrumental sensor response pattern of *Makgeolli* differed clearly according to the rice cultivar and blending ratio (Figure 4). *Makgeolli* C100 exhibited the highest intensities of sourness (AHS = 6.7) and umami (NMS = 8.4), whereas *Makgeolli* G100 and B100, both derived from single glutinous rice cultivars, showed low umami scores but relatively strong responses from the sweetness- and bitterness-related sensors (ANS and SCS, respectively). The blended sample C60G40 exhibited relatively low sourness and medium-to-high umami, indicating an instrumental response pattern characterized by relatively lower sourness-related response and medium-to-high umami-related response. In contrast, C80G20 exhibited relatively high sourness and saltiness (CTS) along with the simultaneous activation of multiple other taste sensors, suggesting a more complex multi-sensor response pattern in the E-tongue measurements.

E-tongue responses appeared to be influenced by certain physicochemical properties and the free amino acid composition. In *Makgeolli* C100, B100, and C80G20, which exhibited strong sourness, the total acid contents were also high, consistent with the interpretation that the AHS sensor response is associated with total acidity. On the other hand, in *Makgeolli* G100, the combined levels of umami-related amino acids, such as glutamic acid and aspartic acid, were relatively high, but the umami score was low, indicating that the umami response of the E-tongue cannot be explained by amino acid composition alone. Overall, compared with non-glutinous rice, glutinous rice (cultivars *Gureumchal* and *Baekokchal*) tended to enhance sweetness but showed no clear advantage in terms of umami. Yu et al. [53] also reported that although *Hwangjiu* produced from glutinous and japonica rice cultivars showed slight differences in the contents of several sweet- and umami-related amino acids, E-tongue analysis revealed no significant differences in sweetness or umami attributes. Similarly, our findings suggest that the effects of rice cultivar and blending ratio on taste may vary depending on the fermentation conditions and environment.

This study systematically evaluated overall *Makgeolli* taste using an E-tongue, assessing a range of non-glutinous and glutinous rice cultivars and cultivar blending ratios. Most notably, C60G40 simultaneously exhibited moderate acidity, relatively high umami-related response, and relatively strong sweetness-related sensor response, suggesting that C60G40 may be a promising formulation for further validation, which should be confirmed by trained sensory panel evaluation.

## 4. Conclusions

This study showed that the physicochemical properties, volatile aromatic compounds, and E-tongue-derived taste profiles of *Makgeolli* were influenced by the rice cultivar and blending ratios used during fermentation. *Makgeolli* produced from *Gureumchal* (glutinous rice) was associated with higher levels of ethyl esters linked to fruity aroma profiles, potentially through acyl-CoA–related metabolism. The *Gureumchal*-based samples also showed higher free amino acid levels, which may contribute to higher ANS (sweetness-related) responses, suggesting that this cultivar could be a promising candidate raw material for creating *Makgeolli* with fruity flavor profiles. In contrast, the non-glutinous rice samples exhibited higher production of fusel alcohols and acetate esters, which may be related to acetyl-CoA and Ehrlich-type metabolism, differing from the trend observed in glutinous rice samples. In blended samples, the E-tongue results showed that the C60G40 exhibited the most balanced taste characteristics, demonstrating that flavor can be systematically designed through controlled combinations of rice cultivars. Because only a limited set of cultivars/blends and a single fermentation protocol were tested, these findings may not fully generalize to other cultivars or processing conditions. In addition, E-tongue outputs are instrumental indicators and should be validated using a trained sensory panel. Overall, *Makgeolli* quality appears to be influenced by multiple factors, including fermentation conditions as well as rice starch molecular structure, saccharification efficiency, yeast metabolic pathways, and cultivar-specific characteristics. Our findings also highlight the practical potential of *Gureumchal* in *Makgeolli* production cultivar and provide baseline data for establishing a raw material strategy for developing *Makgeolli* with targeted flavor profiles.

## Figures and Tables

**Table 1:** Physicochemical characteristics of *Makgeolli* made using different rice cultivars and blend ratios.

Property	Rice Formulations				
	C100	B100	G100	C80G20	C60G40
Soluble solid (°Brix)	7.20 ± 0.01 ^a^	7.20 ± 0.01 ^a^	6.95 ± 0.01 ^c^	7.00 ± 0.01 ^b^	6.37 ± 0.01 ^d^
Reducing sugar (%)	0.29 ± 0.01 ^c^	0.32 ± 0.01 ^a^	0.22 ± 0.01 ^e^	0.30 ± 0.01 ^b^	0.27 ± 0.01 ^d^
Alcohol content (%)	8.60 ± 0.01 ^b^	8.80 ± 0.01 ^a^	8.40 ± 0.01 ^c^	8.80 ± 0.01 ^a^	8.60 ± 0.01 ^b^
pH	3.23 ± 0.10 ^cd^	3.28 ± 0.01 ^d^	3.33 ± 0.01 ^c^	3.44 ± 0.01 ^b^	3.52 ± 0.01 ^a^
Total acidity (%, *w*/*v*)	0.61 ± 0.01 ^a^	0.59 ± 0.01 ^b^	0.49 ± 0.01 ^d^	0.62 ± 0.01 ^a^	0.56 ± 0.01 ^c^
Organic acid (mg/mL)					
Citric acid	1.33 ± 0.15 ^a^	1.47 ± 0.22 ^a^	1.42 ± 0.15 ^a^	1.30 ± 0.23 ^a^	1.48 ± 0.27 ^a^
Lactic acid	1.96 ± 0.07 ^a^	2.17 ± 0.16 ^a^	2.09 ± 0.13 ^a^	2.10 ± 0.18 ^a^	1.93 ± 0.19 ^a^
Free sugar (mg/L)					
Glucose	116.06 ± 2.18 ^a^	109.21 ± 6.60 ^a^	40.44 ± 2.11 ^c^	77.07 ± 4.20 ^b^	81.10 ± 4.86 ^b^
Fructose	350.65 ± 7.98 ^b^	425.30 ± 18.61 ^a^	247.50 ± 9.24 ^c^	210.48 ± 9.09 ^d^	353.52 ± 15.50 ^b^

Rice formulation abbreviations include C (*Baekjinju*, non-glutinous rice), B (*Baegokchal*, glutinous rice), and G (*Gureumchal*, glutinous rice). All the data were expressed as the mean ± SD (*n* = 3). In each row, values followed by different letters differ significantly (*p* < 0.05).

**Table 2:** Free amino acid contents of *Makgeolli* made using different rice cultivars and cultivar ratios.

Taste	Amino Acid	Free Amino Acid Content (mg/L)				
		C100	B100	G100	C80G20	C60G40
Umami	Glu	4.56 ± 0.24 ^ab^	3.41 ± 0.19 ^c^	4.44 ± 0.26 ^b^	4.71 ± 0.27 ^ab^	5.17 ± 0.22 ^a^
	Asp	6.42 ± 0.19 ^a^	4.88 ± 0.37 ^b^	6.73 ± 0.16 ^a^	6.70 ± 0.02 ^a^	6.78 ± 0.24 ^a^
	Subtotal	10.98 ± 0.15 ^b^	8.29 ± 0.19 ^c^	11.17 ± 0.31 ^ab^	11.40 ± 0.26 ^ab^	11.95 ± 0.46 ^a^
Sweet	Pro	21.18 ± 0.64 ^a^	17.23 ± 0.86 ^b^	23.57 ± 1.48 ^a^	22.67 ± 0.29 ^a^	21.33 ± 1.03 ^a^
	Gly	4.84 ± 0.10 ^c^	4.77 ± 0.12 ^c^	4.75 ± 0.29 ^c^	5.90 ± 0.15 ^a^	5.29 ± 0.06 ^b^
	Ala	22.41 ± 1.23 ^a^	17.56 ± 0.56 ^b^	25.19 ± 1.08 ^a^	22.81 ± 0.96 ^a^	23.47 ± 1.80 ^a^
	Thr	6.96 ± 0.30 ^b^	7.08 ± 0.30 ^b^	8.32 ± 0.27 ^a^	7.94 ± 0.44 ^a^	7.79 ± 0.23 ^ab^
	Ser	4.55 ± 0.31 ^b^	4.82 ± 0.33 ^ab^	5.60 ± 0.32 ^a^	5.57 ± 0.51 ^a^	5.22 ± 0.22 ^ab^
	Subtotal	59.95 ± 1.70 ^b^	51.47 ± 0.62 ^c^	67.43 ± 2.07 ^a^	64.89 ± 0.52 ^ab^	63.11 ± 3.05 ^ab^
Bitter	Val	17.05 ± 1.52 ^bc^	15.30 ± 0.43 ^c^	19.81 ± 0.45 ^a^	18.67 ± 0.84 ^ab^	19.16 ± 0.85 ^ab^
	Met	1.56 ± 0.05 ^a^	1.25 ± 0.10 ^c^	1.36 ± 0.07 ^bc^	1.40 ± 0.06 ^abc^	1.52 ± 0.01 ^ab^
	Ile	12.35 ± 0.13 ^b^	11.25 ± 0.66 ^b^	14.39 ± 0.95 ^a^	14.26 ± 0.60 ^a^	12.78 ± 0.66 ^ab^
	Leu	12.97 ± 0.80 ^ab^	10.72 ± 0.49 ^b^	14.46 ± 0.48 ^a^	13.82 ± 0.60 ^a^	14.62 ± 0.70 ^a^
	Phe	7.53 ± 0.21 ^ab^	6.78 ± 0.20 ^ab^	8.07 ± 0.32 ^a^	8.24 ± 0.34 ^a^	7.65 ± 0.41 ^a^
	Lys	6.16 ± 0.44 ^ab^	5.60 ± 0.32 ^b^	6.19 ± 0.08 ^ab^	6.62 ± 0.34 ^a^	6.46 ± 0.07 ^a^
	His	3.30 ± 0.10 ^ab^	3.07 ± 0.07 ^b^	3.59 ± 0.21 ^a^	3.55 ± 0.09 ^a^	3.48 ± 0.27 ^ab^
	Arg	9.49 ± 0.55 ^ab^	7.38 ± 0.35 ^c^	10.28 ± 0.43 ^a^	9.65 ± 0.62 ^ab^	9.01 ± 0.27 ^b^
	Subtotal	70.4 ± 2.5 ^bc^	61.4 ± 1.5 ^d^	78.2 ± 0.6 ^a^	76.2 ± 1.6 ^ab^	74.7 ± 0.7 ^b^
Neutral	Tyr	6.09 ± 0.31 ^cd^	5.80 ± 0.41 ^d^	6.81 ± 0.18 ^bc^	7.11 ± 0.09 ^bc^	7.79 ± 0.25 ^a^
Unflavored	Cys	1.34 ± 0.06 ^a^	0.96 ± 0.08 ^b^	1.31 ± 0.01 ^a^	1.34 ± 0.05 ^a^	1.36 ± 0.06 ^a^
Total		148.7 ± 0.8 ^c^	127.9 ± 1.0 ^d^	164.9 ± 2.2 ^a^	161.0 ± 2.1 ^ab^	158.9 ± 2.7 ^b^

Rice formulation abbreviations include C (*Baekjinju*, non-glutinous rice), B (*Baegokchal*, glutinous rice) and G (*Gureumchal*, glutinous rice). All the data were expressed as the mean ± SD (*n* = 3). In each row, values followed by different letters differ significantly (*p* < 0.05).

## Data Availability

The original contributions presented in this study are included in the article. Further inquiries can be directed to the corresponding authors.
